# Supplementary material for: Blood stem cell-forming haemogenic endothelium in zebrafish derives from arterial endothelium
Source: Nat Commun. 2019 Aug 8;10:3577. doi: 10.1038/s41467-019-11423-2 (PMC6687740; doi:10.1038/s41467-019-11423-2)
Supplement: Supplementary file 1 — Supplementary Information [file 41467_2019_11423_MOESM1_ESM.pdf]

Supplementary Information

Blood stem cell-forming haemogenic endothelium in zebrafish derives from arterial endothelium

Bonkhofer et al.

Supplementary figures

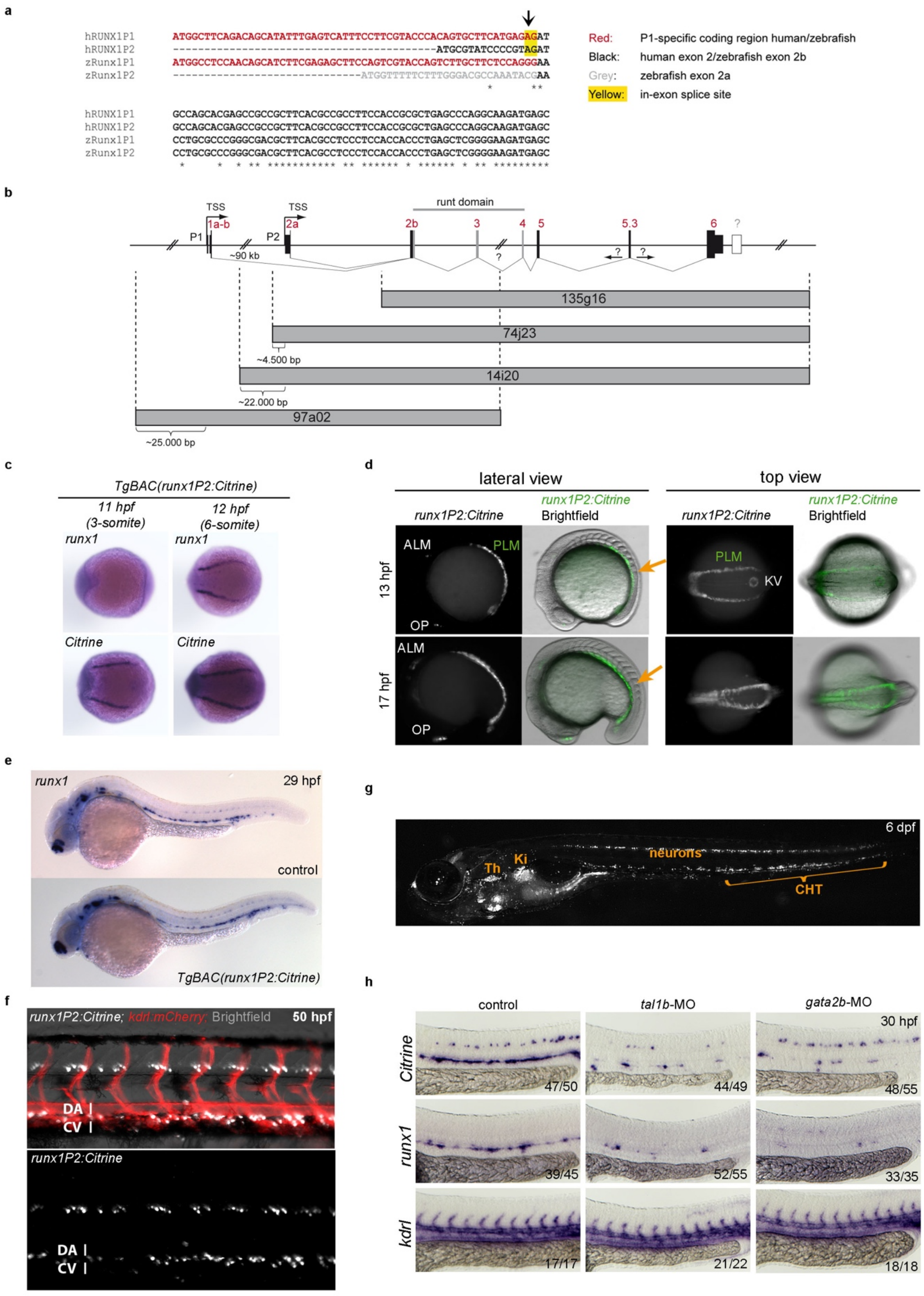

Supplementary Figure 1: Proper regulation of *Citrine* expression in *TgBAC(runx1P2:Citrine)* embryos

(**a**) Multiple sequence alignment between the 5' most bases of the *runx1* alternative promoter products from human (hRUNX1) and zebrafish (zRunx1). P1 specific sequences are shown in red. The zebrafish 2a specific sequence is shown in grey. The in-exon splice site for the human transcript is highlighted in yellow. (**b**) Schematic representation of the zebrafish *runx1* locus and regions covered by each of the previously identified zebrafish BACs[1]. (**c**) ISH for *runx1* and *Citrine* in early *TgBAC(runx1P2:Citrine)* embryos depicting the region of the posterior lateral mesoderm (PLM). (**d**) Microscopic images of Citrine fluorescence focusing on the PLM region. Orange arrows indicate region shown in the top view panel. (**e**) ISH for *runx1* in WT and early *TgBAC(runx1P2:Citrine)* embryos at 29 hpf. (**f**) Fluorescent microscopy image of a 50 hpf double transgenic *TgBAC(runx1P2:Citrine);Tg(kdrl:mCherry)* embryo focusing on the trunk region. (**g**) Representative microscopic image of a 6 dpf *TgBAC(runx1P2:Citrine)* embryo. The region of the thymus (Th), kidney (Ki) and caudal haematopoietic tissue (CHT) are indicated. (**h**) ISH for *Citrine* or *runx1* in the trunk region of embryos injected with MOs targeting *tal1b* or *gata2b*. ISH for *kdrl* controls for integrity of vascular structures.

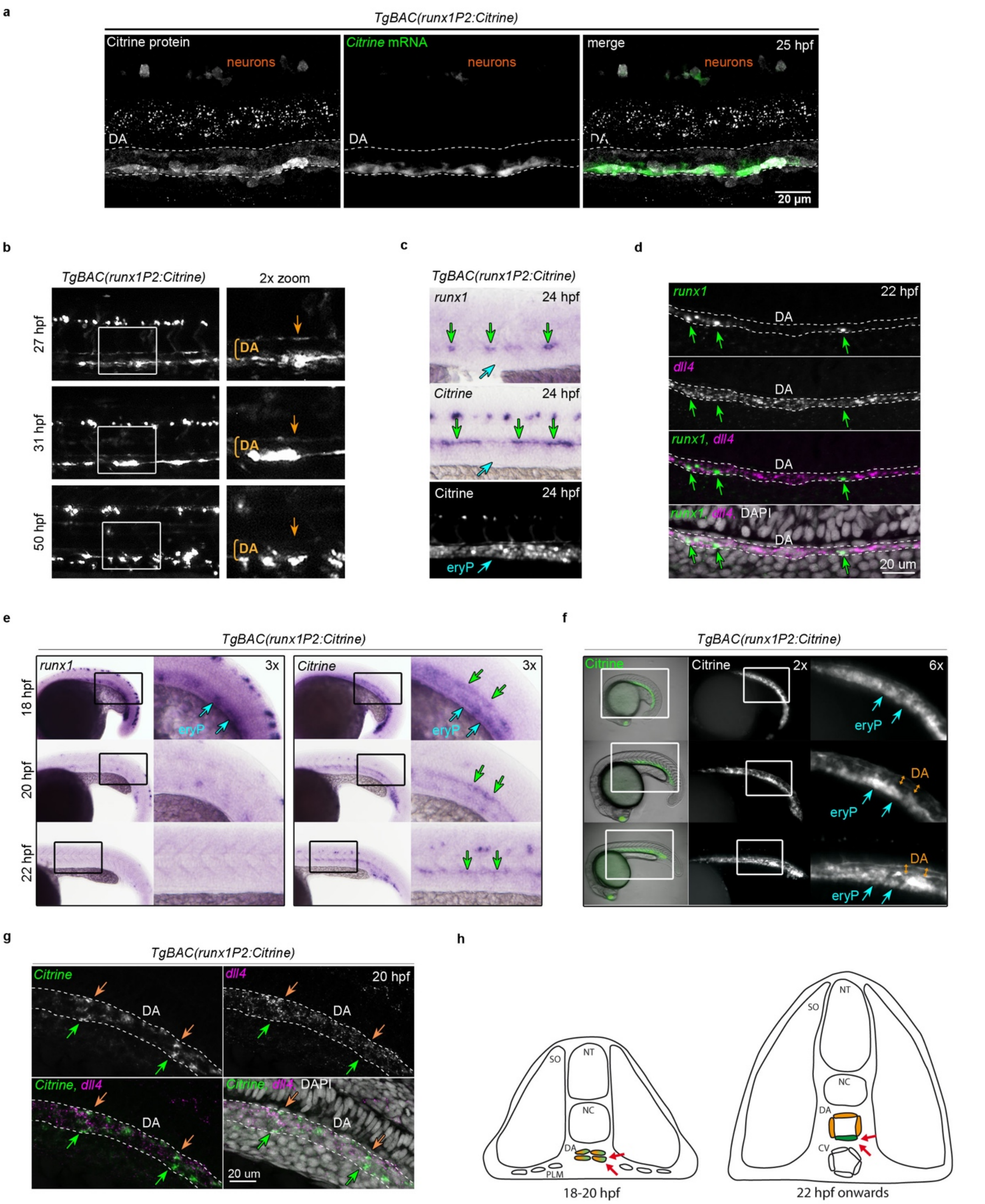

Supplementary Figure 2: Initial activation of *Citrine* expression before DA lumenisation causes carryover of Citrine protein in the aortic roof endothelium

(**a**) Confocal image of a whole mount 25 hpf *TgBAC(runx1P2:Citrine)* embryo immunostained for Citrine protein (white) combined with FISH for *Citrine* mRNA (green). The DA structure is outlined. (**b**) Analysis of *Citrine* transgene and endogenous *runx1* expression in 24 hpf *TgBAC(runx1P2:Citrine)* embryos by ISH (*runx1* and *Citrine*) and fluorescent microscopy (Citrine). Green arrows point to the HE. Blue arrows point to the sub-aortic region containing primitive erythrocytes (eryP). (**c**) Representative fluorescent microscopic images of the DA region from *TgBAC(runx1P2:Citrine)* embryos over time. Image acquisition was performed under identical illumination conditions. The magnifications highlight the boxed regions. Orange arrows point to the DA roof. (**d**) Maximum intensity projection of a representative confocal image after double FISH for *dll4* and *runx1* in the DA region of a 22 hpf embryo. Green arrows point to *runx1*$^+$ cells. (**e**) Expression analysis of *runx1* and *Citrine* during early DA development (18-22 hpf) in *TgBAC(runx1P2:Citrine)* embryos. Left: ISH analysis of *runx1*. Right: ISH analysis of *Citrine*. (**f**) Fluorescent microscopy images of Citrine fluorescence during early DA development (18-22 hpf) in *TgBAC(runx1P2:Citrine)* embryos. For **e** and **f**: blue arrows point to expression in primitive erythrocytes (eryP); green arrows point to the DA/HE region. Orange double-arrows indicate the DA lumen. (**g**) Maximum intensity projection of a representative confocal image of double FISH for *dll4* and *runx1* in the DA region of a 20 hpf embryos. orange arrows point to the ARE and green arrows to the HE. (**h**) Model depicting the DA region of zebrafish embryos before and after lumenisation. DA angioblasts that reach the embryonic midline are initially clustered together. Subsequent lumenisation creates the geometry for a signalling gradient in which the HE (green) stays in the inductive signalling zone (red arrows) driving the up-regulation of haematopoietic

genes. ARE (orange) maintains an arterial programme. CV: cardinal vein; DA: dorsal aorta; NC: notochord; NT: neural tube; SO: somites.

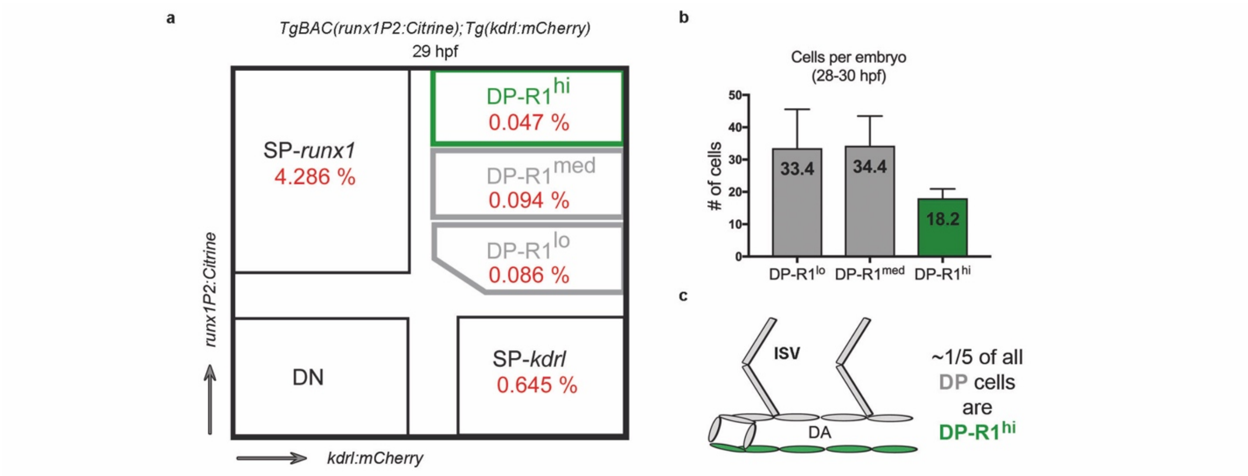

Supplementary Figure 3: Quantification of aortic and haemogenic cells in zebrafish embryos

(**a**) Quantification of the sorted cell populations from double transgenic *TgBAC(runx1P2:Citrine);Tg(kdrl:mCherry)* embryos at ~29 hpf. The numbers represent the proportion of the respective population in relation to the whole embryo (n =4). (**b**) Total cell counts per embryo as detected by FACS for the indicated cell fractions. (**c**) Schematic of the zebrafish DA structure with the HE highlighted in green. Including all cells of the DA and the sprouting ISV, the HE reflects approximately 1/5 of the whole DA structure.

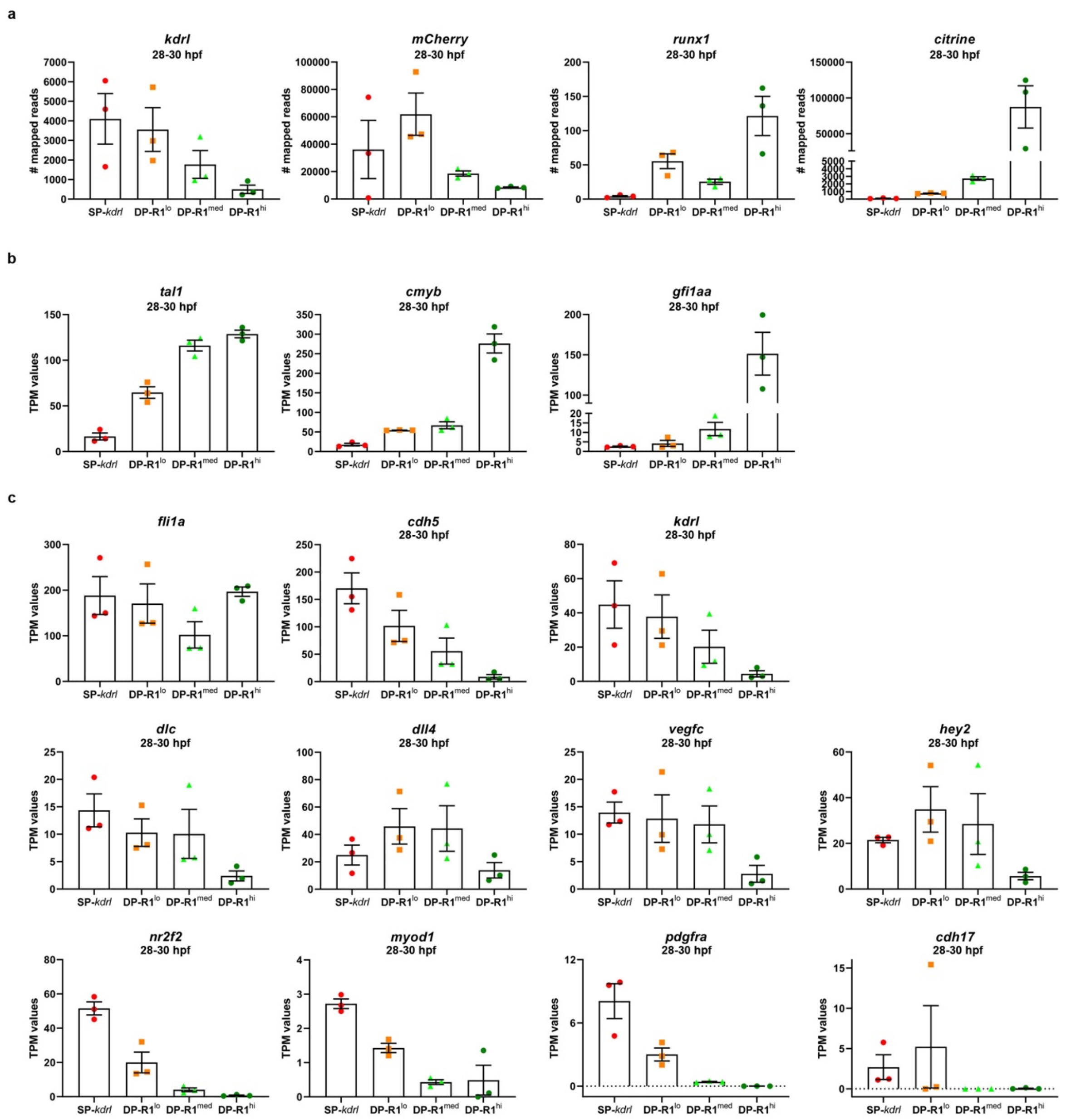

Supplementary Figure 4: Internal control gene analysis after RNAseq in HE, ARE and NAE

(**a**) Number (#) of mapped reads against *mRNA* sequences for: *kdrl*, *mCherry*, *runx1* and *Citrine*. (**b**) TPM value of the haemogenic genes *tal1*, *cmyb* and *gfi1aa* as detected by RNAseq in the in detected populations. (**c**) TPM value analysis of further genes including endothelial genes (*fli1a*, *cdh5* and *kdrl*), arterial genes (*dlc*, *dll4*, *vegfc* and *hey2*), the venous gene *nr2f2*, as well as the myotome marker *myod1*, the sclerotome/neural crest marker *pdgfra* and the pronephros marker

cdh17. TPM values for *myod1*, *pdgfra* and *cdh17* are low suggesting minimal contamination by other tissues. Error bars represent the SEM.

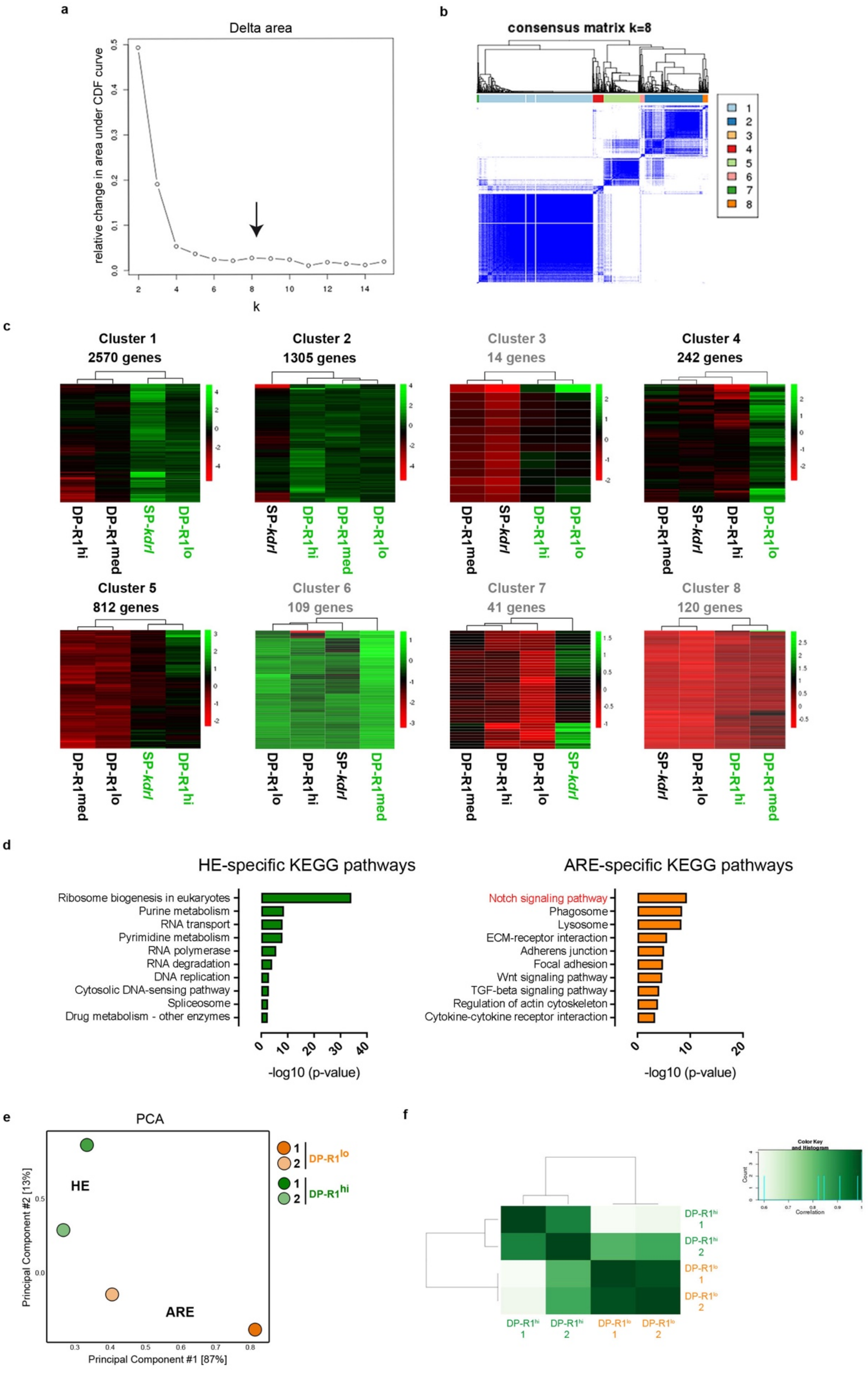

a
Delta area
relative change in area under CDF curve
k
b
consensus matrix k=8
1
2
3
4
5
6
7
8
c
Cluster 1
2570 genes
DP-R1hi
DP-R1med
SP-kdrl
DP-R1lo
Cluster 2
1305 genes
SP-kdrl
DP-R1hi
DP-R1med
DP-R1lo
Cluster 3
14 genes
DP-R1med
SP-kdrl
DP-R1hi
DP-R1lo
Cluster 4
242 genes
DP-R1med
SP-kdrl
DP-R1hi
DP-R1lo
Cluster 5
812 genes
DP-R1med
DP-R1lo
SP-kdrl
DP-R1hi
Cluster 6
109 genes
DP-R1lo
DP-R1hi
SP-kdrl
DP-R1med
Cluster 7
41 genes
DP-R1med
DP-R1hi
DP-R1lo
SP-kdrl
Cluster 8
120 genes
SP-kdrl
DP-R1lo
DP-R1hi
DP-R1med
d
HE-specific KEGG pathways
Ribosome biogenesis in eukaryotes
Purine metabolism
RNA transport
Pyrimidine metabolism
RNA polymerase
RNA degradation
DNA replication
Cytosolic DNA-sensing pathway
Spliceosome
Drug metabolism - other enzymes
-log10 (p-value)
ARE-specific KEGG pathways
Notch signaling pathway
Phagosome
Lysosome
ECM-receptor interaction
Adherens junction
Focal adhesion
Wnt signaling pathway
TGF-beta signaling pathway
Regulation of actin cytoskeleton
Cytokine-cytokine receptor interaction
-log10 (p-value)
e
PCA
HE
ARE
Principal Component #2 [13%]
Principal Component #1 [87%]
DP-R1lo
DP-R1hi
f
Color Key and Histogram
Count
Correlation
DP-R1hi 1
DP-R1hi 2
DP-R1lo 1
DP-R1lo 2

Supplementary Figure 5: Determination of the genetic programmes in HE, ARE and NAE

(**a**) Delta area plot of consensus clustering identified an optimal setting of k = 8 clusters from cumulative distribution function (CDF) (**b**) Consensus clustering matrix for k = 8 of RNA-seq data from all 4 analysed endothelial sub-populations. Each row represents one gene. The intensity in blue represents the frequency at which two genes were observed to cluster together. Cluster numbers are indicated at the right. (**c**) Heatmaps for each cluster resulting from consensus clustering with the respective dendrogram on top. Gene expression levels are normalized by Z-score transformation across all data sets. The numbers of genes for each cluster are indicated. Clusters with >240 genes are shown in black. Smaller clusters are shown in grey. Cell populations with high levels of gene expression for each respective cluster are shown in green. (**d**) KEGG-pathway analysis on HE-specific (green) and ARE-specific (orange) gene-sets. Shown are the top 10 enriched terms. Notch signalling related terms are highlighted in red. (**e**) Principle component analysis (PCA) for DP-R1lo and DP-R1hi open chromatin regions as identified by ATAC-seq. (**f**) Correlation plot for DP-R1lo and DP-R1hi ATAC-seq replicates.

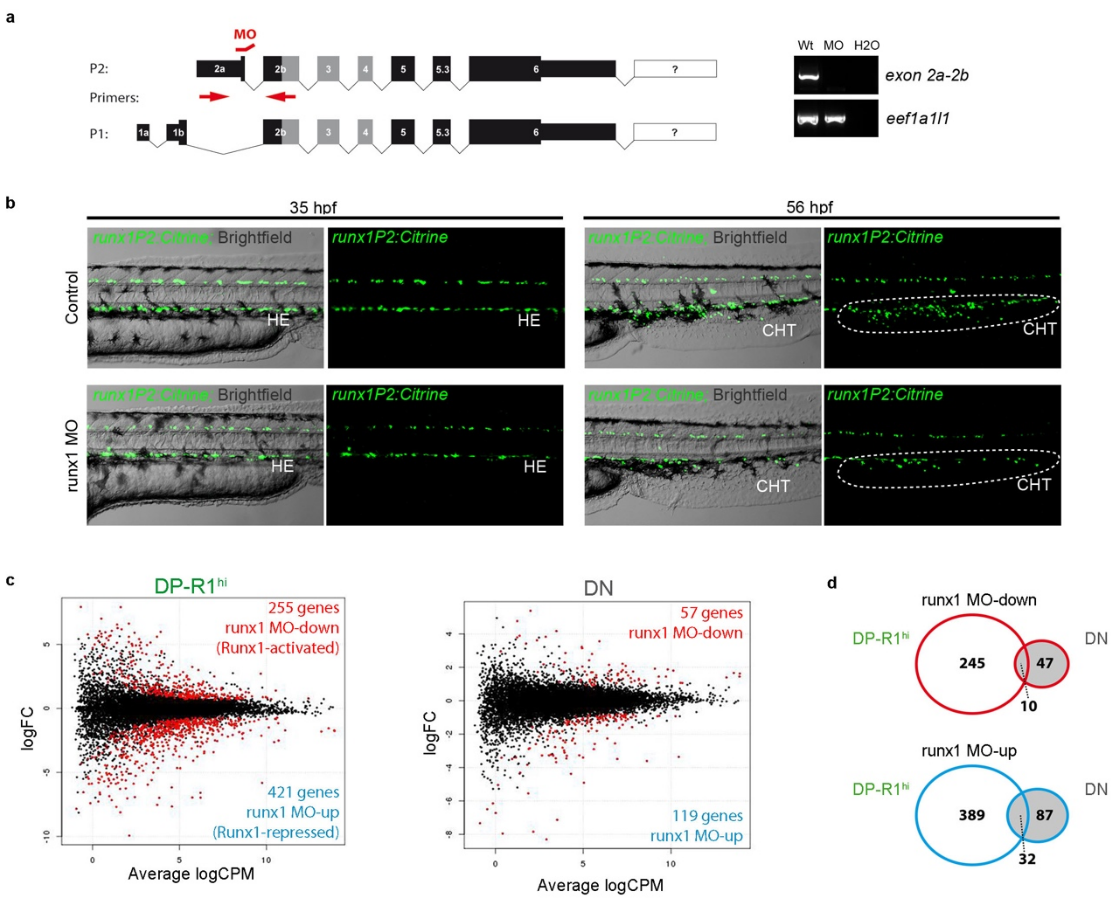

Supplementary Figure 6: Set-up of a *runx1* MO based gene expression screen

(**a**) Left: Schematic of the alternative transcripts derived from the two alternative promoters P1 and P2 and the binding site for the used P2-specific *runx1* splice-MO. Right: Typical agarose gel for the analysis of the splice-MO functionality using indicated primers. (**b**) Fluorescent microscopic images of *TgBAC(runx1P2:Citrine)* embryos either un-injected (control) or *runx1* MO injected. Left: Showing the trunk region including the DA of 35 hpf embryos. Right: Showing the caudal haematopoietic tissue (CHT) region of 56 hpf embryos. (**c**) Smear plot result of differentially expressed gene analysis (DEG) between *runx1* MO and control embryos. Left: DEG analysis for cells of the DP-R1^{hi} gate showing significantly down-regulated (red; Runx1-activated) and up-regulated (blue; Runx1 repressed) genes. Right: DEG analysis for cells of the DN gate

showing significantly down-regulated (red) and up-regulated (blue) genes. (**d**) Gene list intersection between DEG detected in cells of the DP-R1^{hi} and DN gates after *runx1* MO injection. Top: Intersection between genes down-regulated due to *runx1* MO injection in both populations. Bottom: Intersection between genes down-regulated due to *runx1* MO injection in both populations.

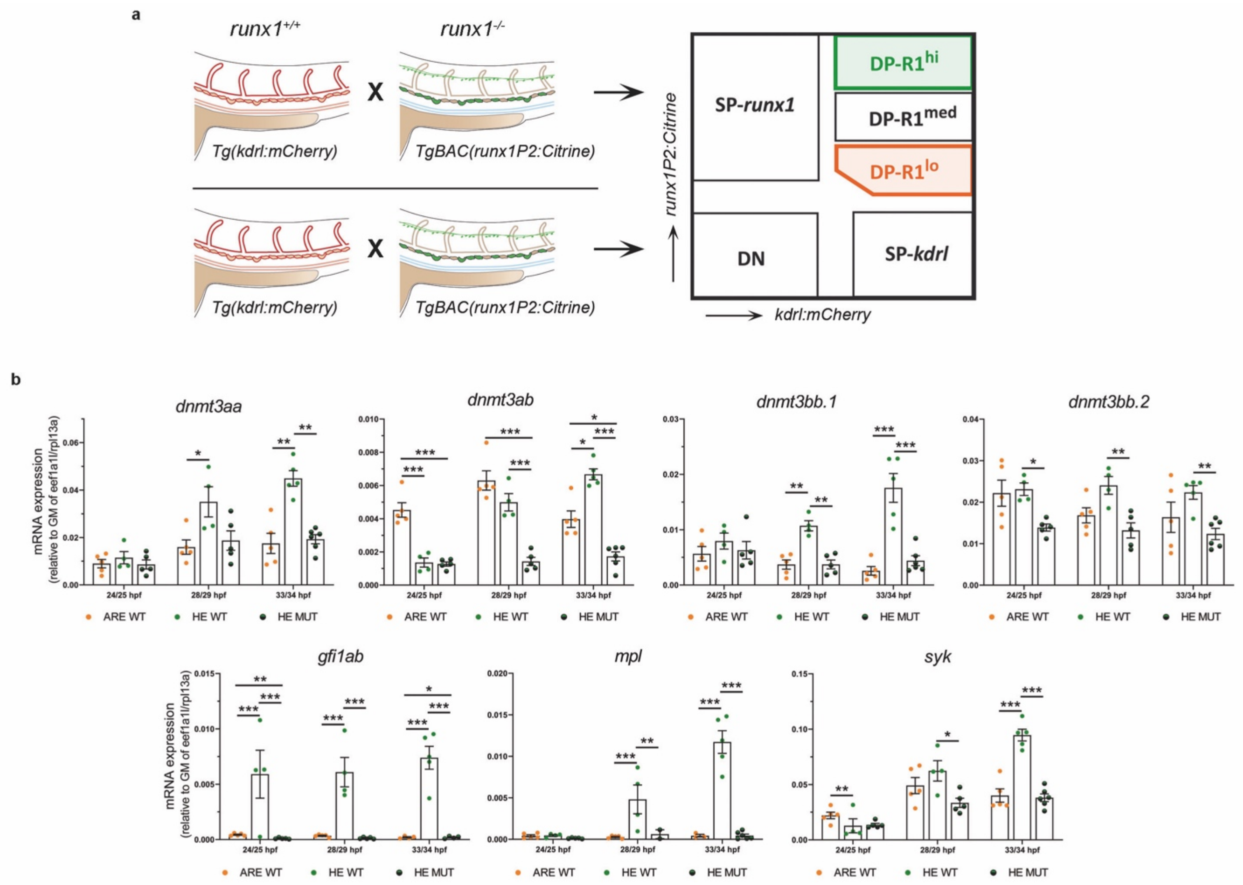

Supplementary Figure 7: Gene expression in Runx1 mutants validates Runx1 targets identified by RNA-seq

(**a**) Illustration of the experimental set-up to study gene expression of potential Runx1 targets by qRT-PCR in *runx1*$^{+/+}$ and *runx1*$^{-/-}$ mutants using the BioMark platform. Transgenic *TgBAC(runx1P2:Citrine)* and *Tg(kdrl:mCherry)* reporter lines were crossed on a homozygous *runx1*$^{-/-}$ background. Double transgenic offspring of either *runx1*$^{+/+}$ or *runx1*$^{-/-}$ in-crosses were used to isolate cells of the indicated populations for subsequent gene expression analysis by qRT-PCR. (**b**) qRT-PCR gene expression analysis of potential Runx1 targets (*dnmt3aa*, *dnmt3ab*, *dnmt3bb.1*, *dnmt3bb.2*, *gfi1ab*, *mpl* and *syk*) in the HE and ARE of *runx1*$^{+/+}$ (WT) and the HE of *runx1*$^{-/-}$ mutant (MUT) embryos. Graphs show the mean of detected expression levels relative to the geometric mean (GM) of the 2 housekeeping genes *eef1a1l* and *rpl13a*. n = 5 independent

biological experiments for WT embryos and n = 6 independent biological experiments for MUT embryos. Error bars represent the SEM. 2-way ANOVA; * = $p<0.05$; ** = $p<0.01$; *** = $p<0.001$.

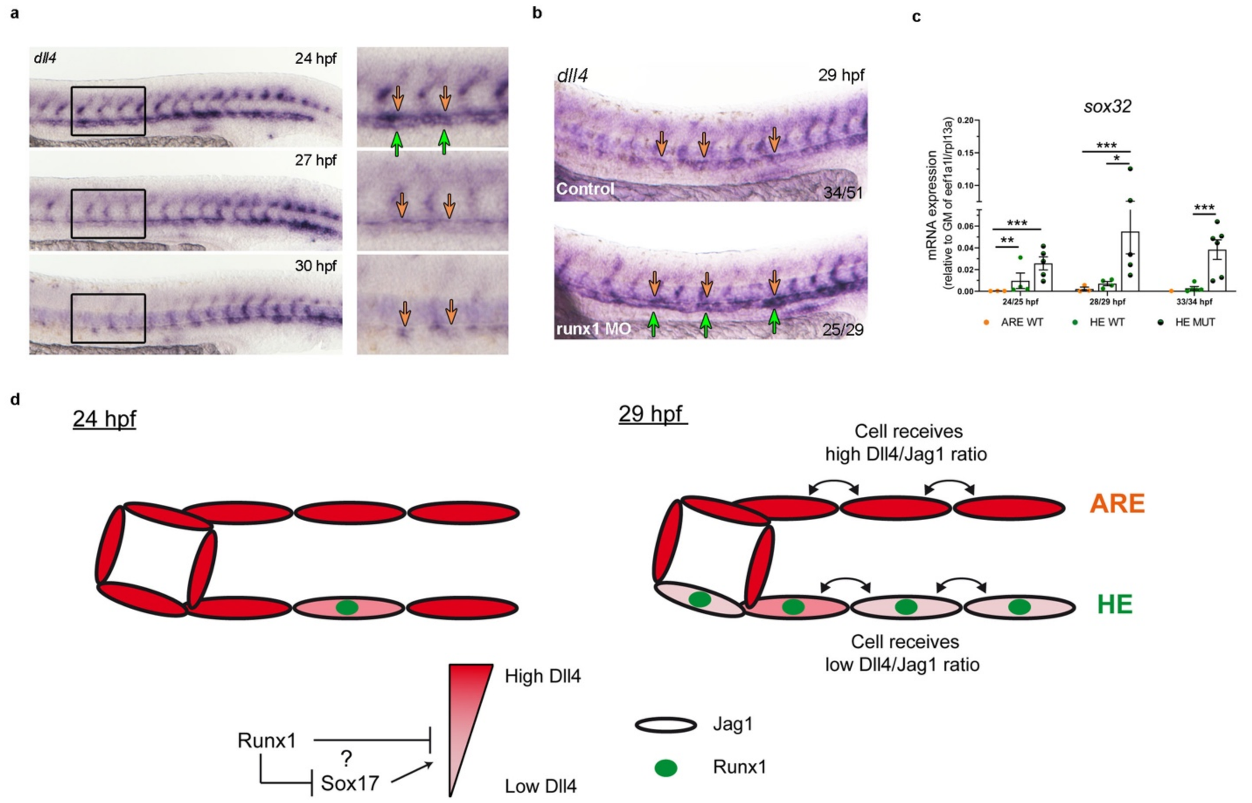

Supplementary Figure 8: Down-regulated expression of *dll4* in HE accounts for a lower *Dll4* to *jag1a* ratio driving haemogenic specification

(**a,b**) Spatial analysis of gene expression by ISH. Green arrows point to the HE. Orange arrows point to the DA roof. (**a**) Time line analysis of *dll4* during definitive haematopoiesis in the DA region. The magnifications highlight the boxed regions. (**b**) ISH analysis of *dll4* in *runx* MO and control embryos. (**c**) qRT-PCR gene expression analysis of *sox32* in the HE and ARE of *runx1*$^{+/+}$ (WT) and the HE of *runx1*$^{-/-}$ mutant (MUT) embryos. Graph shows the mean of detected expression levels relative to the geometric mean (GM) of the 2 housekeeping genes *eef1a1l* and *rpl13a*. n = 5 independent biological experiments for WT embryos and n = 6 independent biological experiments for MUT embryos. Error bars represent the SEM. 2-way ANOVA; * = $p<0.05$; ** = $p<0.01$; *** = $p<0.001$. (**d**) Model depicting the dynamics of NOTCH ligands *jag1a*

and *dll4* expression in the zebrafish DA. Up-regulation of *runx1* in the HE causes a repression of *dll4*, either directly or through the repression of the positive input Sox17, whereas *jag1* expression stays uniform. Despite an early non-synchronous initiation of *runx1* in the HE, over time such cells are neighboured by further cells with low *dll4* expression. Consequently, HE-specific repression of *dll4* leads to a low Dll4/Jag1 ratio for such cells shown to be required for further haematopoietic differentiation, whereas cells in the ARE retain a higher Dll4/Jag1 ratio.

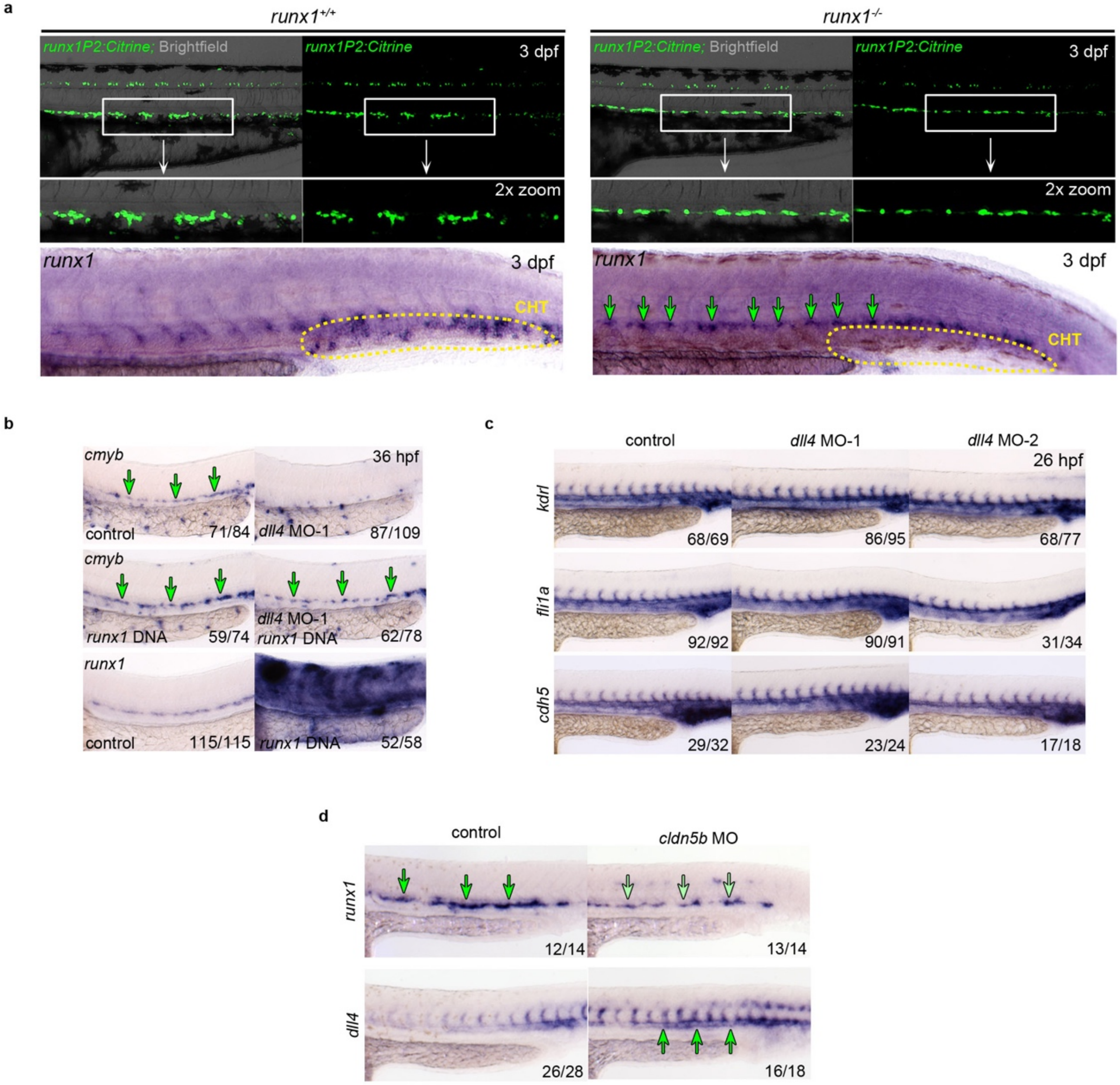

Supplementary Figure 9: Arterial identity is a prerequisite for aortic HE

(**a**) Top: Representative fluorescent microscopy images highlighting the DA region of 3 dpf *TgBAC(runx1P2:Citrine)* embryos either on a *runx1*$^{+/+}$ or *runx1*$^{-/-}$ genetic background. Magnifications highlight the boxed areas. Bottom: ISH analysis of *runx1* in the trunk region of 3 dpf *runx1*$^{+/+}$ and *runx1*$^{-/-}$ embryos. Green arrows point to expression in the HE. The region of the caudal haematopoietic tissue (CHT) is indicated. (**b**) Top and middle: ISH analysis of *cmyb* in *dll4* MO-1 and control embryos co-injected with or without a *runx1* over-expression construct. Green arrows point to the HE. Bottom: Expression of *runx1* in control and *runx1* over-expression embryos. (**c**) ISH analysis of endothelial genes *kdrl, fli1a* and *cdh5* in control and *dll4* MO embryos. Experiment was performed with two different MOs. (**d**) ISH analysis of runx1 and *dll4* expression in control and *cldn5b* MO embryos.

	Zebrafish gene names	Cluster (C) 1,2,3,4 (Li, Y. et al 2014)[2]	HECs and HSCs (Kartalaei, P. S. et al. 2015)[3]	RUNX1 ChIP binding; ChIP-seq studies see Legend	Legend
1	angpt1	C1	HSCs	A, B	**A:**<br>RUNX1 ChIP in HCP7 cells (Wilson, N. et al. 2010)[4]<br>**B:**<br>RUNX1 and RUNX1/ETO ChIP in Kasumi-1 cells (Li, Y. et al. 2016)[5]<br>**C:**<br>RUNX1 ChIP in Kasumi-1 cells and two t(8;21) patients (Ptasinska, A. et al 2012)[6]
2	ankrd10a	C4	/	/	
3	cyfip2	C1	/	/	
4	dnmt3ab	C4	HSCs	A, B, C	
5	dnmt3ba	C1	HSCs	B	
6	dnmt3bb.1	C1	HSCs	B	
7	dnmt3bb.2	C1	HSCs	B	
8	ezrb	C4	/	/	
9	gfi1ab	C1	HSCs	B, C	
10	irf1b	C1	/	A, B, C	
11	mpl	C1	/	A	
12	mybbp1a	C1	/	/	
13	pbrm1l	C2	/	B	
14	pdxka	C1	/	B	
15	pik3cd	C1	/	B, C	
16	pik3r1	C1	/	A, B	
17	selp	C1	/	A	
18	stmn1a	C1	/	A, B	
19	syk	C1	HSCs	A, B	
20	wdhd1	C2	HSCs	B	
21	zgc:92791	C1	/	/	
22	znf296	C1	HSCs	/	

Supplementary Table 1: List of high-confidence Runx1 target genes specific to the HE

Genes identified as HE-specific and Runx1-activated were intersected and cross-compared to published RNA-seq and Runx1 ChIP-seq data sets to identify genes with an evolutionarily conserved expression and the potential to be bound by Runx1. Green: Gene is present in the respective data set. Grey: Gene is absent in the respective data set. C1/C2/C4: Cluster 1/2/4 as defined by the Li et al. (2014)[2] study. Haemogenic endothelial cells (HEC) and haematopoietic stem cells (HSCs) are defined by the Kartalaei et al. (2015)[3] study.

Supplementary references

1. Lam, E. Y. N. *et al.* Zebrafish runx1 promoter-EGFP transgenics mark discrete sites of definitive blood progenitors. *Blood* **113,** 1241–1249 (2009).

2. Li, Y. *et al.* Inflammatory signaling regulates embryonic hematopoietic stem and progenitor cell production. *Genes Dev.* **28,** 2597–2612 (2014).

3. Kartalaei, P. S. *et al.* Whole-transcriptome analysis of endothelial to hematopoietic stem cell transition reveals a requirement for Gpr56 in HSC generation. *J. Exp. Med.* **212,** 93–106 (2015).

4. Wilson, N. K. *et al.* Combinatorial transcriptional control in blood stem/progenitor cells: Genome-wide analysis of ten major transcriptional regulators. *Cell Stem Cell* **7,** 532–544 (2010).

5. Li, Y. *et al.* Genome-wide studies identify a novel interplay between AML1 and AML1/ETO in t(8;21) acute myeloid leukemia. *Blood* **127,** 233–242 (2016).

6. Ptasinska, A. *et al.* Depletion of RUNX1/ETO in t(8;21) AML cells leads to genome-wide changes in chromatin structure and transcription factor binding. *Leukemia* **26,** 1829–1841 (2012).
