# Supplementary material for: Blood stem cell-forming haemogenic endothelium in zebrafish derives from arterial endothelium
Source: Nat Commun. 2019 Aug 8;10:3577. doi: 10.1038/s41467-019-11423-2 (PMC6687740; doi:10.1038/s41467-019-11423-2)
Supplement: Supplementary file 2 — Description of Additional Supplementary Files [file 41467_2019_11423_MOESM2_ESM.pdf]

Description of Additional Supplementary Files

File Name: Supplementary Data 1
Description:The genes in each cluster after consensus clustering. Related to Supplementary Fig. 4.

File Name: Supplementary Data 2
Description: List of differentially expressed genes between DP-R1^{lo} and DP-R1^{hi} populations. Shown are the gene count values of the replicates, the logFC and statistical information. Related to Fig. 3.

File Name: Supplementary Data 3
Description: List of differential ATAC-peaks between DP-R1^{lo} and DP-R1^{hi} populations. Provided are the genomic annotation and the next gene locus.

File Name: Supplementary Data 4
Description: List of differentially expressed genes between wild type (WT) and runx1 MO samples for both, DP-R1^{hi} and double negative (DN) populations. Shown are the gene count values of the replicates, the logFC and statistical information. Additionally, the list of true off-targets defined as common down- and up-regulated genes for both populations is provided as well. Related to Fig. 4.

File Name: Supplementary data 5
Description: List of primers and morpholinos (MO) used in this study.
