# Supplementary material for: Blood stem cell-forming haemogenic endothelium in zebrafish derives from arterial endothelium
Source: Nat Commun. 2019 Aug 8;10:3577. doi: 10.1038/s41467-019-11423-2 (PMC6687740; doi:10.1038/s41467-019-11423-2)
Supplement: Supplementary file 9 — Reporting Summary [file 41467_2019_11423_MOESM9_ESM.pdf]

nature research

Corresponding author(s): Roger Patient
Rui Monteiro

Last updated by author(s): Jun 11, 2019

Reporting Summary

Nature Research wishes to improve the reproducibility of the work that we publish. This form provides structure for consistency and transparency in reporting. For further information on Nature Research policies, see Authors & Referees and the Editorial Policy Checklist.

Statistics

For all statistical analyses, confirm that the following items are present in the figure legend, table legend, main text, or Methods section.

n/a	Confirmed	
☐	☒	The exact sample size (*n*) for each experimental group/condition, given as a discrete number and unit of measurement
☐	☒	A statement on whether measurements were taken from distinct samples or whether the same sample was measured repeatedly
☐	☒	The statistical test(s) used AND whether they are one- or two-sided<br>*Only common tests should be described solely by name; describe more complex techniques in the Methods section.*
☒	☐	A description of all covariates tested
☐	☒	A description of any assumptions or corrections, such as tests of normality and adjustment for multiple comparisons
☐	☒	A full description of the statistical parameters including central tendency (e.g. means) or other basic estimates (e.g. regression coefficient) AND variation (e.g. standard deviation) or associated estimates of uncertainty (e.g. confidence intervals)
☐	☒	For null hypothesis testing, the test statistic (e.g. *F*, *t*, *r*) with confidence intervals, effect sizes, degrees of freedom and *P* value noted<br>*Give P values as exact values whenever suitable.*
☒	☐	For Bayesian analysis, information on the choice of priors and Markov chain Monte Carlo settings
☒	☐	For hierarchical and complex designs, identification of the appropriate level for tests and full reporting of outcomes
☒	☐	Estimates of effect sizes (e.g. Cohen's *d*, Pearson's *r*), indicating how they were calculated

Our web collection on statistics for biologists contains articles on many of the points above.

Software and code

Policy information about availability of computer code

Data collection	Sequencing data (RNAseq and ATACseq) was obtained from (Illumina HiSeq4000 and NextSeq machine, respectively) in FastQ format and mapped/processed /analysed using the software listed below
Data analysis	STAR aligner 2.4.2a<br>BWA aligner 0.7.12<br>Trimmomatic 0.32<br>subread 1.6.2<br>Homer 3.0<br>R 3.4.1-newgcc<br>R bioconductor packge<br>R diffBinding package<br>R pheatmap package<br>R ConsensusClusterPlus package

For manuscripts utilizing custom algorithms or software that are central to the research but not yet described in published literature, software must be made available to editors/reviewers. We strongly encourage code deposition in a community repository (e.g. GitHub). See the Nature Research guidelines for submitting code & software for further information.

Data

Policy information about availability of data

All manuscripts must include a data availability statement. This statement should provide the following information, where applicable:

- Accession codes, unique identifiers, or web links for publicly available datasets
- A list of figures that have associated raw data
- A description of any restrictions on data availability

All data generated or analysed during this study are included in this published article (and its supplementary information files). Accession codes for RNAseq and

ATACseq are GSE132259 [https://www.ncbi.nlm.nih.gov/geo/query/acc.cgi?acc=GSE132259] and GSE132258 [https://www.ncbi.nlm.nih.gov/geo/query/acc.cgi?acc=GSE132258], respectively.

Field-specific reporting

Please select the one below that is the best fit for your research. If you are not sure, read the appropriate sections before making your selection.

☒ Life sciences ☐ Behavioural & social sciences ☐ Ecological, evolutionary & environmental sciences

For a reference copy of the document with all sections, see nature.com/documents/nr-reporting-summary-flat.pdf

Life sciences study design

All studies must disclose on these points even when the disclosure is negative.

Sample size	For RNAseq and ATACseq experiments a minimum of 3 samples was determined as sufficient due to the homogeneous genetic background of the zebrafish population and the high numbers of fish were pooled for each independent experiment. For confirmatory gene expression experiments using the Biomark platform sample size was increased to 5-6 independent experiments.
Data exclusions	No data were excluded for the gene expression analysis. For ATACseq triplicates were analysed for consistency by PCA and correlation analysis and one outlying replicate has been removed for each, DP-R1hi and DP-R1lo.
Replication	Differential gene expression analysis by RNAseq was based on runx1-morpholino technology. Functionality of the morpholino in each independent experiment was verified by PCR and phenotype observation (see Supplementary Fig. 6). Reproducibility of the experimental findings was verified ISH and Biomark experiments in runx1-mutants.
Randomization	For each independent RNAseq experiments (Wt and morpholino) embryos from several clutches were pooled. To control for potential batch effects, every single clutch was split in half; while one half of the clutch remained unmanipulated, the other half was injected with the runx1 morpholino. Afterwards, injected and non-injected embryos from different clutches were pooled accordingly for FACS sorting.
Blinding	Investigators were not blinded

Reporting for specific materials, systems and methods

We require information from authors about some types of materials, experimental systems and methods used in many studies. Here, indicate whether each material, system or method listed is relevant to your study. If you are not sure if a list item applies to your research, read the appropriate section before selecting a response.

Materials & experimental systems

n/a	Involved in the study	
☐	☒	Antibodies
☒	☐	Eukaryotic cell lines
☒	☐	Palaeontology
☐	☒	Animals and other organisms
☒	☐	Human research participants
☒	☐	Clinical data

Methods

n/a	Involved in the study	
☒	☐	ChIP-seq
☐	☒	Flow cytometry
☒	☐	MRI-based neuroimaging

Antibodies

Antibodies used	Antibodies for in situ hybridization: Anti-digoxigenin-AP, Fab fragments (11093274910, SIGMA) and Anti-Fluorescein-POD, Fab fragments (11426346910, SIGMA)
Validation	All validation of the antibodies used can be found in the maufacturer's website

Animals and other organisms

Policy information about studies involving animals; ARRIVE guidelines recommended for reporting animal research

Laboratory animals	Zebrafish (danio rerio), males and females, AB strain. Tg(kdrl:Hsa.HRAS-mCherry), dll4sa9436 mutants and runx1W84X mutants. Fish were analyzed during embryogenesis (mostly around 24-36 hours post fertilization).
Wild animals	Study did not involve wild animals
Field-collected samples	Study did not involve samples collected from the field

Ethics oversight	All experiments were performed under a Project license approved by the Home Office, UK, under the Animals (Scientific Procedures) Act 1986.

Note that full information on the approval of the study protocol must also be provided in the manuscript.

Flow Cytometry

Plots

Confirm that:

☒ The axis labels state the marker and fluorochrome used (e.g. CD4-FITC).

☒ The axis scales are clearly visible. Include numbers along axes only for bottom left plot of group (a 'group' is an analysis of identical markers).

☒ All plots are contour plots with outliers or pseudocolor plots.

☒ A numerical value for number of cells or percentage (with statistics) is provided.

Methodology

Sample preparation	To isolate sufficient amounts of RNA for the next generation sequencing analysis, at least 3000 cells per population were collected. Zebrafish embryos were staged to 28-30 hours post fertilization, pre-sorted for the presence of fluorescent reporters using the Olympus stereo microscope MVX10 and collected in low binding microcentrifuge tubes (SafeSeal Microcentriguge Tubes, Sorenson; Cat#39640T). Yolk was removed using 116 mM NaCl/2.9 mM KCl/5 mM HEPES (with freshly added 1mM EDTA) deyolking buffer. Cells were dissociated using collagenase/trypsin buffer (20 mg collagenase in 0.05% Trypsin with EDTA in 1x HBSS solution). Reaction was stopped in 1x HBSS/10 mM HEPES/0.25% BSA. Dissociated cells were passed through a 40 µm cell strainer and re-suspended in appropriated volume (~10 µl per embryo; ~3-7x10^6 cells per ml) of 1x HBSS/10 mM HEPES/0.25% BSA with HoechstDEAD (33258; Invitrogen) (1:4000 dilution). FAC-sorting was carried out by the WIMM Flow Cytometry Facility using the BD FACS Aria Fusion system. Cells were either directly sorted into RLT buffer (RNA isolation) or into 1x HBSS/10 mM HEPES/0.25% BSA buffer for subsequent preparation of ATAC-seq libraries.
Instrument	BD FACS Aria Fusion system
Software	FACSDiva v8.0.1 Software
Cell population abundance	SP-runx1: 4.286%; SP-kdrl: 0.645%: DP-R1hi: 0.047%; DP-R1med: 0.094%: DP-R1lo: 0.086%
Gating strategy	We first sorted for live/dead cells using a Hoechst(DEAD) dye, then excluded doublets. From these we set the gates for negative and positive Citrine or mCherry fluorescence with wildtype and single Citrine or mCherry transgenics as single-colour controls. To identify the gating strategy for the DP-cells, we split the DP-gate into three sub-gates of similar height to select for a DP-R1hi population with best enrichment of haemogenic marker genes.

☒ Tick this box to confirm that a figure exemplifying the gating strategy is provided in the Supplementary Information.
